# Continuous Stripping with Dense Carbon Dioxide

**DOI:** 10.1021/acsomega.3c06087

**Published:** 2023-11-29

**Authors:** Márton Kőrösi, Petra Kántor, Péter Bana, Edit Székely

**Affiliations:** † Department of Chemical and Environmental Process Engineering, Budapest University of Technology and Economics, Műegyetem rakpart 3, Budapest H-1111, Hungary; ‡ Richter Gedeon NyRt., Gyömrői út 19-21, Budapest H-1103, Hungary

## Abstract

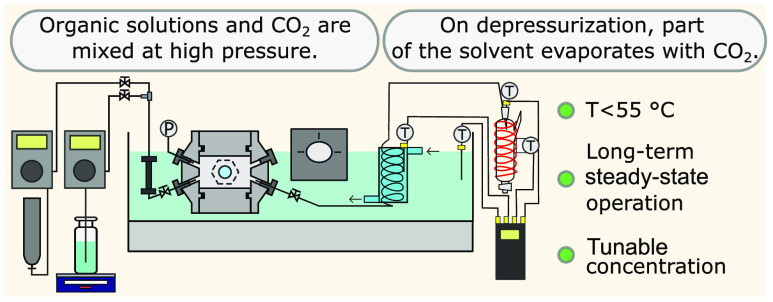

The integration of flow chemistry into continuous manufacturing requires efficient, controllable, and continuous methods for the concentration of diluted solutions on relatively small scales. The design and application examples of a new continuous solvent removal process are presented. The continuous stripping method employing dense carbon dioxide is based on the formation of homogeneous mixtures of dilute organic solutions of the target molecules with a large excess of carbon dioxide at temperatures as low as 35 °C and pressures around 10 MPa. Subsequent pressure reduction results in the quick release of carbon dioxide and vaporization of a significant fraction of the organic solvent. The concentration of the solute in the separated liquid phase can be up to 40 times higher than in the feed. Among the many controllable process parameters, the most significant ones are the mass–flow rate ratio of carbon dioxide to the feed and the temperature of the phase separator. By careful setting of the operational parameters, the degree of concentration enhancement may be accurately controlled. The new apparatus—despite consisting of laboratory equipment and being built in a fume hood—could easily support pilot-scale synthetic flow chemistry, being a continuous, efficient alternative to thermal concentration methods.

## 1 Introduction

**Figure 1:**
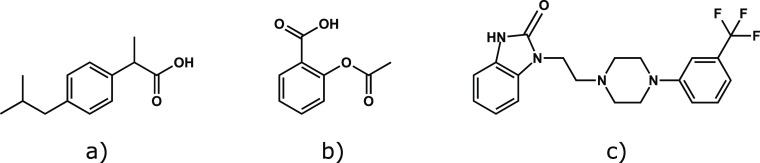
Chemical structures of ibuprofen (a), acetylsalicylic acid (b), and flibanserin (c), the model molecules chosen for developing the presented concentration process and used in its prolonged operational test.

Continuous manufacturing processes are widely applied in large-scale chemical industries such as oil refineries and petrochemical factories. In such cases, voluminous production can be conducted with a steady-state operation and constant product quality. The advantages and the easy automation of continuous industrial apparatuses may also benefit the fine-chemical/pharmaceutical industries.^1^ Due to the smaller required scales, especially in producing active pharmaceutical ingredients and research and development purposes, microreactor technologies are gaining popularity.^2,3^ The possibility of automated parameter screening and straightforward scale-up are attractive features, as well as the possibility of implementing innovative techniques such as microwave heating or photochemical reactions.^4^ Continuous flow chemistry research also closely foreshadows the circumstances of industrial applications more closely. Hazardous or highly exothermic reactions also require fewer considerations in flow reactors.^5^ The small volume and large specific surface area of such reactors mean more efficient heat transfer and fewer dangerous materials than batch tank reactors with similar annual production scales. Rapid heat transfer might also facilitate otherwise slow reactions, and the small volumes make it safe to perform reactions even in pressurized systems. Catalytic and telescoped reactions can also be conducted.^6^ While we do not aim to give a comprehensive view of the available flow chemical methods interesting for producing active pharmaceutical ingredients (APIs), numerous reviews are available in the literature.^4,7−9^ The flow chemical syntheses of ibuprofen, acetylsalicylic acid, and flibanserin (their structures are shown in Figure 1), our selected APIs, have also been developed.^10−14^

For example, continuous processing in the pharmaceutical industry needs several steps following efficient flow-chemical synthesis. Chromatographic purification of the APIs is required between the synthesis and the production of solid dosage forms. In such a case, after purification, the obtained diluted solution of the API must typically be concentrated. Although continuous separation processes capable of concentration enhancement, like distillation or membrane separation, are well known in large-scale industrial manufacturing, their scale-down to the scale of flow-chemical processes^15,16^ is not obvious. In addition, the thermal sensitivities of most APIs may be critical at distillation, while concentrated solutions may easily lead to the fouling of membranes.

Carbon dioxide is a well-known alternative solvent having various applications.^17^ Despite being primarily known as a greenhouse gas, carbon dioxide as process fluid or solvent is green and environmentally benign, and it is also generally regarded as safe (GRAS) solvent. It is mainly used at high pressure or in a supercritical state, where its physical-chemical properties (for example, density and viscosity) depend on pressure and temperature relatively sharply (at least in the vicinity of the critical values).^18^

We aimed to design a technique attachable to a flow-chemical synthesis and achieve controllable concentration enhancement of dilute organic solutions, even for the formation of saturated solutions of the model APIs. Instead of downscaling an existing industrial process, our apparatus was specifically designed to operate at the small scale required. The current article presents the first results of a new concentration enhancement procedure operating under steady-state conditions at mild temperature using pressurized carbon dioxide as a dissolved stripping agent.

To investigate both simple and more complex solvent mixtures and different solute concentrations, we studied the applicability of the process on three different examples of API solutions. Ibuprofen in ethyl acetate was selected to develop the process, fine-tune the equipment, and determine the main process parameters to control and for the “proof-of-principle” study. Acetylsalicylic acid in ethyl acetate containing ethanol as a minor component was selected as a realistic flow of chemical synthetic product stream. Compared to the ibuprofen solution study, the investigation with acetylsalicylic acid solutions aimed to understand the effects of the solute and other minor components, if any. The solution of flibanserin in a four-component solvent mixture is a realistic feed stream that one obtains with a continuous purification coupled after the flow chemical synthesis. This system is presented to show the longer-term production stability of the setup.

## 2 Continuous Concentration of the Solutions of Well-Known APIs

**Figure 2:**
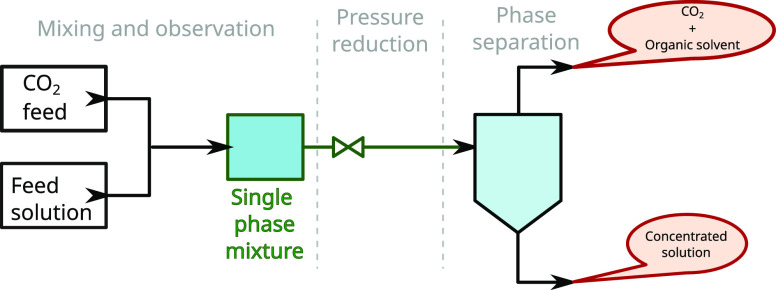
Simplified scheme of the high-pressure concentration device and process. The feed solution and pressurized carbon dioxide form a homogeneous mixture. The mixture is separated into two phases after pressure reduction.

The experimental apparatus is schematically depicted in Figure 2. In the process, carbon dioxide is mixed with an organic solution of a target molecule under high pressure. Subsequent depressurization leads to vapor/liquid phase separation with the pronounced presence of volatile components in the vapor phase.

In the case of ibuprofen, the feed solution contained 0.012 mass fraction of the API in ethyl acetate.

The feed solution of acetylsalicylic acid was prepared according to the solvent system used in its flow synthesis^13^ consisting of the API in a mass fraction of 0.0026 m/m, 0.04 m/m of ethanol, and 0.957 m/m of ethyl acetate.

In the case of flibanserin, the crude API stream exiting the continuous-flow reactor system^14^ can be purified using an extraction-based procedure, after which the API is found in the upper phase of the two-phase mixture, in which a connected continuous stripping apparatus can then concentrate. This process employs a more complex mixture containing multiple solvents, which is modeled in this study by combining the solvents used in the synthesis-purification system and dissolving the API in the upper phase of the resulting two-phase mixture. Cyclohexane, isopropyl acetate, methanol, and water were mixed in 3:7:4:6 volumetric ratio. After equilibration, the two-phase system was separated at room temperature. Flibanserin was dissolved in 1 mg/mL concentration in the upper phase (consisting of cyclohexane, isopropyl acetate, methanol, and water in approximately 35:55:8:2 volumetric ratio). After following the recipe, the mass fraction of the solute in the solvent mixture was around 0.0015.

A Jasco PU-980 Intelligent HPLC pump supplies the feed solution with a flow rate of 0.1–2 mL/min in our apparatus. A Jasco PU-1580-CO2 chromatographic pump delivers carbon dioxide at a flow rate of 0.5–5 mL/min at the given pressure and −4 °C temperature in the cooled pump head. If not noted differently, 1/16 in. tubing with 0.02 in. standard wall thickness and other standard high-pressure Swagelok accessories are installed. Mixing the two liquids is ensured in a tempered conventional 1/16 in. tee union with 0.05 in. internal diameter. After the tee union, 30 cm-long 1/16 in. tubing is installed, and then the mixture enters a tempered view cell of 12 mL in volume. During all measurements, we visually confirmed that the mixture formed a single phase, as noted in Figure 2. The pressure is maintained by exploiting the pressure drop on a capillary of 0.005 in. internal diameter and approximately 1 m length. Most of the capillary is wound up into a coil and tempered. At the end of the capillary, a jet forms in the tempered glass-made atmospheric phase separator of 100 mL.

For the startup procedure, first, the water bath of the view cell and the capillary was tempered, and data logging is started. Then, pure carbon dioxide is pumped through the system with a volumetric flow rate of around 4 mL/min. This allows pressure buildup and prevents the filling of the high-pressure vessel with the organic solvent. When steady pressure with carbon dioxide is reached, the organic solvent (first without a solute to avoid precipitation upon mixing) is introduced into the system. The volumetric flow rates of the pumps are then set to their desired values during the experiment. The organic solvent feed flow is necessary before switching on the PID temperature controller of the phase separator as the latent heat demand of the evaporation of the organic solvent stabilizes its temperature by steadily cooling the internal wall of the heated vessel. Once steady pressure temperature values were achieved in all measurement points, the solvent feed was substituted with the dilute solution of the processed API. The steady state was reached in approximately twice the average residence time. Production may start at this point.

During the parametric studies discussed in this article, the conditions (feed flow rates and temperatures) were kept constant for at least a further 60 min. Samples were collected as described below. When enough samples were taken, selected process parameter(s) were modified, and the new steady-state operation was typically reached after 15–20 min. Steady-state condition was always confirmed by the mass flow rate of the liquid product.

For the shut-off procedure, the feed API solution is switched to a pure solvent for cleaning to avoid API precipitation in the equipment. After suitable washing (appr. 30 min), the solvent feed is stopped, and the equipment is flushed for an additional approx. 30 min with CO_2_ at 4 mL/min flow rate. Depressurization occurs when CO_2_ flow is stopped.

During operation, liquid samples completely drawn from the phase separator were collected to monitor the process: the liquid product obtained during a preset time interval (5 to 10 min) was a single sample. Their mass as a solution was immediately measured. The dry mass of the solute in the concentrate was determined after the evaporation of the solvents from the samples with a Biotage TurboVap LV atmospheric tempered multisample evaporator (by stripping the remaining organic solvent from the samples using nitrogen).

The degree of concentration enhancement of sample *i*, marked by η_*i*_, is the ratio of the mass fraction of the API in the liquid product (*x*_*i*_) over the mass fraction of the API in the feed solution (*x*_0_):
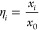


A balance monitored the mass flow rate of the feed solution, while the pumps displayed the volumetric flow rates of the solvents. The density of carbon dioxide () was taken from The NIST Chemistry Webbook^19^ at −4 °C (head temperature of the carbon dioxide pump and the actual pressure in the pump). It was used to calculate the mass flow rate of CO_2_ from the volumetric flow rate. The solvent ratio (*R*) was defined as the ratio of carbon dioxide and organic solution mass flow rate.

## 3 Results and Discussion

The small-scale flow system was constructed with several variable operational parameters. These include the pressure of mixing, the three different regulated temperatures (mixing, pressure release, and phase separation sections), and the two mass flow rates of the feed solvent, carbon dioxide, and pressure. In the following paragraphs, we discuss these process parameters’ effects.

### 3.1 The Effect of the Solvent Ratio

**Figure 3:**
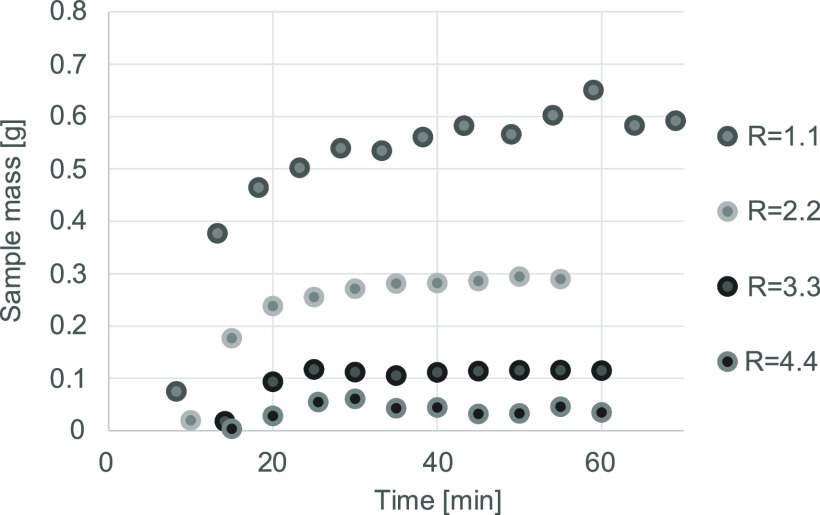
Masses of the samples taken every 5 min while operating with the ethyl acetate solution of ibuprofen. Points are plotted to the middle of the time frame in which the samples were collected.

The solubility of ibuprofen in ethyl acetate is very high, providing us with the possibility of experimenting with operation techniques and process parameters without having too many concerns about the precipitation of the drug. The experiments shown in Figure 3 were conducted at 10 MPa and 35 °C in the mixing section. The diagram serves as a demonstration of the steady-state operation of the apparatus. The liquid product was completely drained from the separation vessel every 5 min. The masses of these samples—closely related to the mass flow rate of the concentrate—are plotted against time in Figure 3. In these measurements, the volumetric flow rate of the organic solution was 1 mL/min, while the volumetric flow rate of carbon dioxide was varied. The *R* values of the specific experiments are shown in the diagram legend.

After about 20 to 30 min of operation, depending on the overall mass flow rate, a steady state was reached in all cases. This steady state can be maintained indefinitely, as shown in Section 3.3.

**Figure 4:**
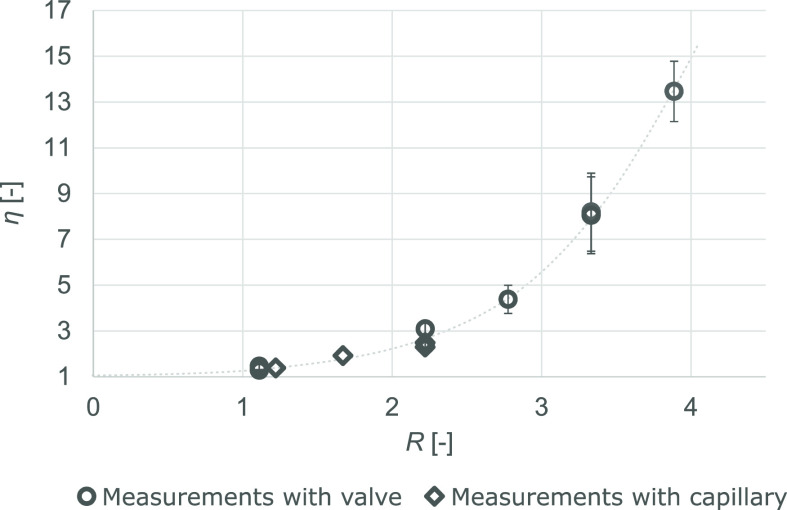
Concentration of ibuprofen in ethyl acetate. The average degree of concentration enhancement values are plotted against the mass flow rate ratios (*R* = mass flow rate of CO_2_/mass flow rate of the feed solution), accompanied by the corrected standard deviation among the samples of each measurement. The dashed line is a guide to the eye; it does not result from a mathematical fitting but suggests an exponential correlation.

Additional experiments were conducted, maintaining the same pressure and temperature (approximately 10 MPa and 35 °C in the mixing section). Also, the feed solution’s volumetric (and thus mass) flow rate was kept constant (at 1 mL/min). The effect of the ratio of the mass flow rates on the degree of concentration enhancement can be observed in Figure 4.

Two data sets are plotted in the diagram. Circular markers show the results obtained by using a manual needle valve to regulate pressure in the apparatus. It is very observable that switching the valve to a capillary (results plotted with diamonds) did not significantly affect the degree of concentration enhancement at any given solvent ratio. The advantage of the capillary lies mainly in the operational stability of the apparatus. In addition, these latter measurements followed the others by almost 1 year and were conducted by a different person. In the diagram, every point marks the average degree of concentration enhancement value calculated based on several (6 to 15) samples from a steady-state measurement. The corrected standard deviation values among individual samples are included for each measurement conducted with a manual valve serving as the pressure regulator. In the case of the lowest setting, the margins are not visible, and the corrected standard deviation values of the two runs are approximately 0.0311 and 0.157. The uncertainty of the process grows with an increasing solvent ratio. However, it would not cause any problems in the longer run, as in a real production scenario, the target variable is a long-term average degree of concentration enhancement in the steady state. It was expected at the beginning that the ratio of the mass flow rates of carbon dioxide and the organic (dilute) solution would influence the portion of volatile solvent taken away. Because of conducting all the experiments at a constant organic solvent flow rate, the total mass flow rate increases as *R* increases. This also leads to a significant decrease in the average residence time in the apparatus. However, if the mixture can become homogeneous before its depressurization, then its average residence time is not a decisive factor.

The effect of the solvent ratio on the degree of concentration enhancement was similar when the dilute solution of acetylsalicylic acid was concentrated: the higher the solvent ratio, the higher the degree of concentration enhancement. The solvent ratio settings were altered within uninterrupted experimental runs in the acetylsalicylic acid study, meaning that once at pressure–temperature values at all controlled locations of the unit—CO_2_ flow rate—solution flow rate setting, the steady state was achieved, the experiment was continued for 30 to 60 min, then new set values were adjusted, and the transition to a different steady-state operational point was observed. Detailed data analysis confirmed that stable operating points were achieved regardless of the previous set values. Increasing the solvent ratios increased the corrected standard deviation values among samples at a single operational point.

The experience gathered on ibuprofen and acetylsalicylic acid, supported by some preliminary experimental runs, was successfully used to determine and set a solvent ratio for the concentration of flibanserin, as described in the next section.

### 3.2 The Effect of the Temperature

Temperature is monitored at four different points and regulated at three different points of the apparatus. The temperature effects are discussed in the order in which the medium passes through the respective measurement locations.

The temperature of the mixing section was kept around 35 °C in most experiments, but increasing it to approximately 50 °C did not have any effect on the degree of concentration enhancement. Although providing the latent heat demand of evaporation in this part of the apparatus is tempting, there are certain limitations to the achievable temperature: the thermal stress on the API and the effect of the temperature (and pressure) on the phase equilibria in the mixing section. Regarding the possible thermal stress on the API, we need to consider that the average residence time of the solution is the longest here before it enters the capillary. Hence, in the case of thermally unstable APIs, it may be beneficial to maintain mild conditions. Furthermore, in the mixing section, keeping a homogeneous, single phase is inevitably necessary. Below the crossover pressure,^20^ higher temperature results in a higher pressure needed to maintain a single-phase system. For practical reasons (and following the principles of green chemistry), the lowest possible pressure is preferable and the temperature of the mixing section should not preferably be increased.

These limiting considerations (thermal stress and phase equilibria) must also be considered in the capillary. However, the effect of changing its temperature was important, even if it remained minor, regarding the degree of concentration enhancement itself. As the capillary forms the majority of pressure buildup in the apparatus, changing the temperature in its water bath could be used to fine-tune the operational pressure that can otherwise only be influenced by changing the volumetric flow rate of the solvents.

**Figure 5:**
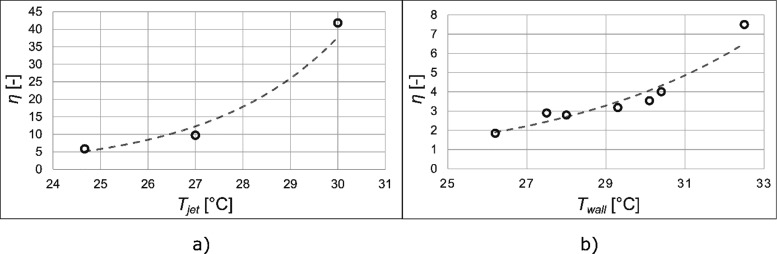
Effect of temperature on the degree of concentration enhancement. (a) Effect of the temperature of the jet exiting the capillary on the example of the ethyl acetate solution of ibuprofen (*R* = 2.2); (b) effect of the temperature of the wall of the separator vessel on the example of the mixed-solvent solution of flibanserin (*R* = 2.57).

The effect of the temperature of the separator was studied in two different systems and resulted in very similar conclusions. In the left panel (Figure 5a), the degree of concentration enhancement of ibuprofen in its ethyl acetate solution is plotted against the temperature of the jet. In these measurements, the separator’s temperature was controlled using this value as feedback. In the right panel (Figure 5b), the effect of the wall temperature of the separator on the concentration of flibanserin dissolved in the mixture of (majorly) cyclohexane and isopropyl acetate is shown. Both cases show an increasing degree of concentration with increasing temperature.

### 3.3 Long-Term Operation under Production Conditions

In the parametric studies described in the previous sections, steady-state operation of the apparatus was always achieved and maintained for a long enough time to use the average degree of concentration enhancement of multiple samples to characterize the process. However, limited time consumption was also an important concern in these runs due to practical reasons. After gaining sufficient operational experience and finding an appropriate combination of operating parameters, we turned our attention to longer experimental runs to prove the real-world applicability of the technique.

Approximately 2 dm^3^ of the solution of flibanserin was prepared and processed. The temperature was continuously monitored and recorded. Apart from the startup period, we experienced that the prototype of the continuous high-pressure concentrator was operating without the intervention of the laboratory personnel for up to over 9 h. Samples were taken to confirm the constant value of the degree of concentration enhancement over time.

**Figure 6:**
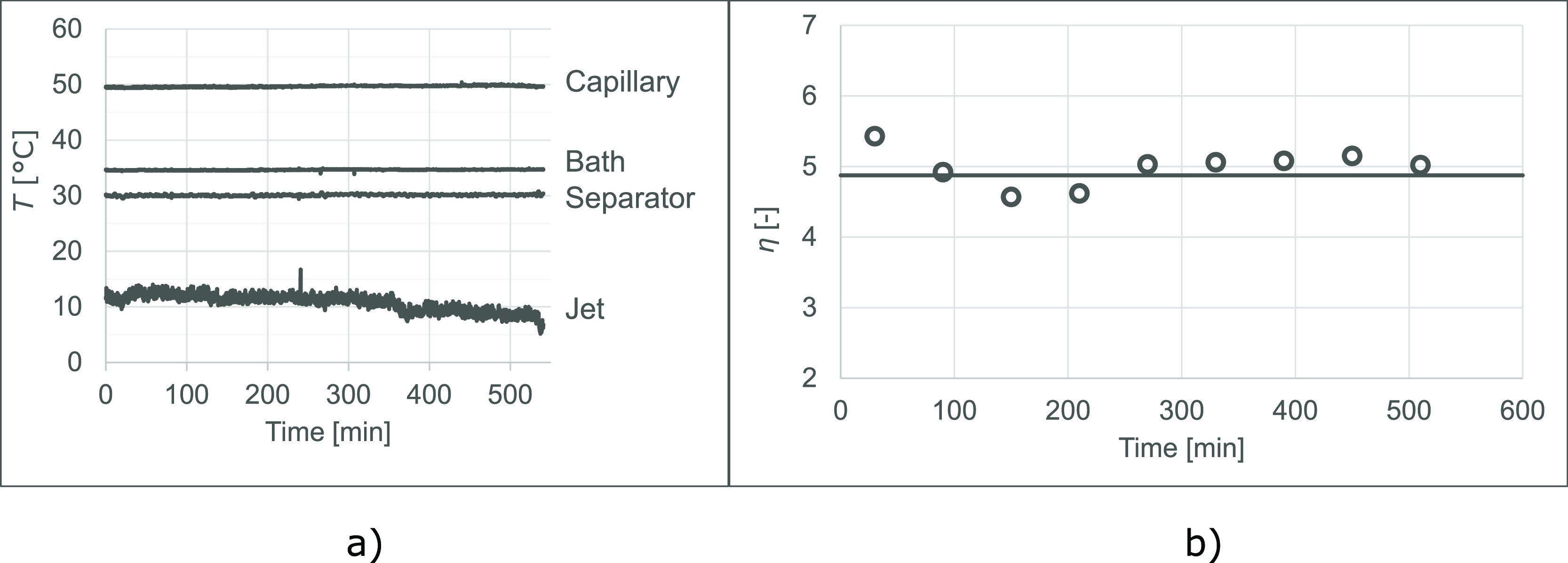
Temperature profile (a) and degree of concentration enhancement (b) of a prolonged concentration run on the example of the solution of flibanserin (*R* = 2.57).

In Figure 6a, the regulated temperature values can be seen at all of the measurement points. Despite the rigorously maintained temperatures, the jet seems to be drifting downward, probably because the thermometer is getting slightly covered in solid deposit throughout the measurement process.

In Figure 6b, spherical markers show the degree of concentration enhancement calculated by comparing sample masses obtained in each hour of operation to the mass of the feeding solution that entered the apparatus during the same period of time. The solid line shows the average degree of concentration enhancement measured on the product solution.

## 4 Conclusions

A new, continuous, low-temperature concentration method was invented to support the continuous flow production of valuable active pharmaceutical agents. A high-pressure mixed solution is produced in the apparatus by adding carbon dioxide to the dilute feed. The partial evaporation of the solution occurred upon pressure reduction. The major advantage of the process is that the concentration of carbon dioxide (and, thus, the phase ratio in the stripping-like process) is not limited when the equipment is operated in the one-phase region of the mixture, while changing the solvent ratio also makes it possible to regulate the extent of concentration. Another significant advantage is that it needs only mild heating to maintain operating temperatures slightly above room temperature, making it possible to process the solutions of thermally unstable components. The viability of the equipment was demonstrated on the ethyl acetate solution of ibuprofen and ethyl acetate/ethanol solution of acetylsalicylic acid and the mixed-solvent solution of flibanserin. The ratio of the mass–flow rates of carbon dioxide and the feed solution and the temperature of the phase separator were the most significant process parameters. The apparatus could operate for more than 9 h in a single run without any decisive intervention from the operator. Hence, despite its laboratory scale, it may be suitable for small-scale production.

## 5 Materials

Acetylsalicylic acid and flibanserin were synthesized in-house as described in the literature.^13,14^ Ibuprofen (racemic) was purchased from Tokyo Chemical Industry with a purity of over 98%. Organic solvents were ordered from Merck with a purity of over 99.5%. Carbon dioxide was supplied by Linde Gas Hungary (purity > 99.5%).
